# In Vitro Evaluation of Speed Sintering and Glazing Effects on the Flexural Strength and Microstructure of Highly Translucent Multilayered 5 mol% Yttria-Stabilized Zirconia

**DOI:** 10.3390/ma17184634

**Published:** 2024-09-21

**Authors:** Ji-In Jeong, Yong-Hoon Kwon, Hyo-Joung Seol

**Affiliations:** Department of Dental Materials, School of Dentistry, Pusan National University, Yangsan-si 626-814, Republic of Korea; stop1989@hanmail.net (J.-I.J.);

**Keywords:** speed sintering, multilayered zirconia, glazing, flexural strength, microstructure

## Abstract

This study aimed to investigate the impact of speed sintering and glazing on the flexural strength and microstructure of multilayered 5 mol% yttria-stabilized (5Y-) zirconia, which remains unknown. Bar-shaped specimens (N = 600) were fabricated from 5Y-zirconia (FX; Ceramill Zolid FX ML, ST; Katana STML) by cutting, polishing, sintering (conventional and speed sintering), and then glazing. A flexural strength test (n = 30/group), field emission scanning electron microscopy (FE-SEM) observation (n = 2/group), and an X-ray diffraction (XRD) study with Rietveld refinement (n = 1/group) were performed. The flexural strength was analyzed using three-way ANOVA and a post hoc Scheffé test. The grain size was analyzed using the Kruskal–Wallis H test and Bonferroni–Dunn post hoc test. Flexural strength slightly decreased in the nonglazed FX after speed sintering (*p* < 0.05). Glazing with and without glazing paste did not affect flexural strength at both sintering speeds (*p* > 0.05). Speed sintering and glazing minimally changed the Weibull modulus and phase fraction, and did not affect grain size (*p* > 0.05). ST had a larger grain size and lower tetragonal phase content than FX and had a lower flexural strength than FX in most groups (*p* < 0.05). Overall, the multilayered 5Y-zirconia is considered suitable for dental application using speed sintering and glazing.

## 1. Introduction

The use of zirconia in dentistry has significantly increased owing to its ease of handling, relatively esthetic appearance compared with metals, and superior mechanical properties compared with other ceramic materials [1,2,3,4,5,6,7]. Zirconia is used in a wide range of dental prostheses, from crowns and bridges to implants [8]. Its exceptional biocompatibility and mechanical strength make it ideal for monolithic implant fabrication [8]. Advanced surface treatments enhance osseointegration, while its superior mechanical properties allow for thin, minimal designs for implants [8].

The addition of stabilizers such as yttria can significantly enhance the esthetics of zirconia [9,10,11,12]. Initially, first-generation zirconia was monolayered; however, multilayered zirconia with improved esthetics has been developed recently. Highly translucent multilayered 5Y-zirconia has an yttria content higher than that of traditional monolayered 3 mol% yttria-stabilized (3Y-) zirconia [13,14,15,16]. This type of zirconia is a third-generation zirconia and can be used as a monolithic restoration without feldspar porcelain veneers [13,14,15,16]. Notably, the multilayered 5Y-zirconia has a color gradient but no yttria gradient, unlike 5Y/3Y-zirconia and other yttria gradient zirconia. As a result, it exhibits an almost uniform composition throughout the layers with minor differences in pigment type or amount [11,17]. The most frequent prosthetic failure of zirconia restorations is caused by chipping and cracking [18]. While 5Y-zirconia displays a lower flexural strength when compared to 3Y-zirconia and 4Y-zirconia, it still demonstrates a higher flexural strength than huge categories of dental silicates such as lithium disilicate, lithium aluminosilicate, and zirconia reinforced lithium silicate [12,19].

The esthetics and flexural strength of zirconia are affected by sintering time and temperature [20,21]. The conventional sintering process for zirconia restorations requires at least 7 to 8 h [9,22]. To address the time-consuming nature of sintering, zirconia manufacturers have proposed a speed sintering protocol [23]. Speed sintering reduces the sintering time, thereby enabling the production of single-day prosthetics. Recent studies show that the use of a speed sintering protocol with various third-generation multilayered zirconia does not negatively impact their esthetic properties [9,22,24]. Moreover, the flexural strength of these zirconia is either increased [9] or remains unaffected by speed sintering [17,22,25,26,27,28].

Zirconia restorations are subjected to an additional heat treatment process called glazing after sintering [29,30]. During glazing, the zirconia surface is coated with a thin layer of glass, which provides several advantages, including filling of surface defects and achieving a more natural appearance [29,31]. The combined impact of speed sintering and subsequent glazing on the flexural strength and microstructure of highly translucent multilayered 5Y-zirconia remains unknown. However, for 3Y-TZP, some studies have focused on the effect of glazing, primarily after conventional sintering, but there are fewer studies on speed sintering [32,33,34,35,36,37]. A previous study reported an increase in the flexural strength of 3Y-TZP following glazing after conventional sintering [32]; however, most researchers have reported a decrease in flexural strength following glazing after conventional sintering [33,34,35,36,37]. One of these reports noted that glazing with a glazing material decreased the flexural strength of conventionally sintered 3Y-TZP (inCoris TZI, Prettau, Zirlux FC), whereas simulated-glazing without a glazing material did not decrease its flexural strength [36]. Speed-sintered and then glazed 3Y-TZP (inCoris TZI C) exhibited a decrease in flexural strength compared with conventionally sintered and glazed specimens; however, because the study did not include a control group that only underwent sintering, whether the difference in flexural strength was caused by speed sintering or glazing was unclear [38]. For 5Y-zirconia, there are few reports on this topic. 5Y-zirconia is a type of highly translucent material and typically multilayered. Thus, the impacts of speed sintering and glazing on the flexural strength of 5Y-zirconia may differ from those of 3Y-TZP. While the impact of glazing on the flexural strength of conventionally sintered 5Y-zirconia has been investigated and found to be negligible [39], the combined impact of speed sintering and glazing on the flexural strength of 5Y-zirconia remains unknown. The zirconia glazing process involves applying glaze paste and firing (heat treatment), which may increase the thickness owing to glazing layer. To exclude the effect of the increased thickness resulting from the glazing layer, this study investigated the impact of speed sintering and glazing, both with and without glazing paste, on the flexural strength and associated microstructure of multilayered 5Y-zirconia. The null hypothesis is that speed sintering and glazing with and without glazing paste do not affect the flexural strength and microstructure of multilayered 5Y-zirconia.

## 2. Materials and Methods

### 2.1. Sample Preparation

**Figure 1:**
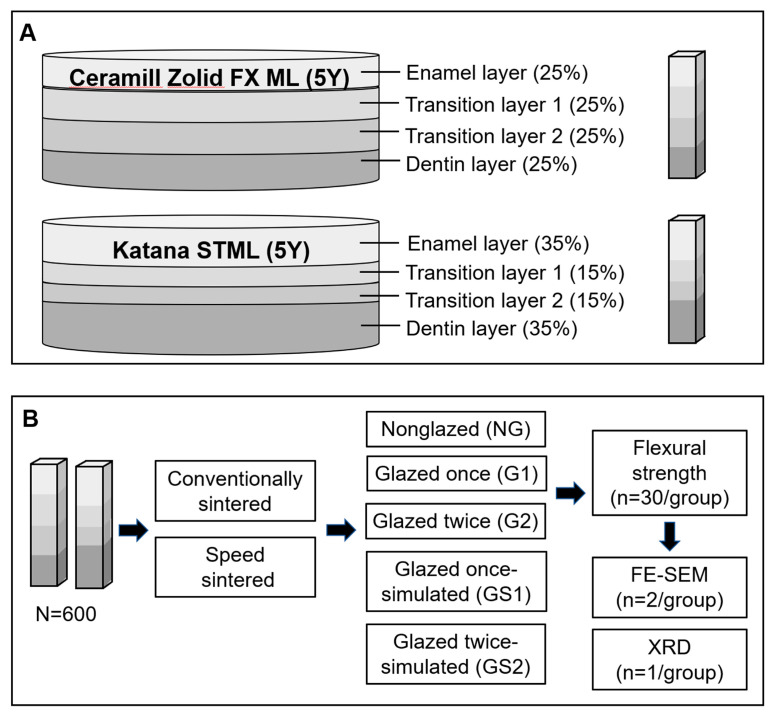
Shapes of Ceramill Zolid FX ML/Katana STML blocks, and fabricated specimen (**A**); study design flowcharts (**B**).

**Figure 2:**
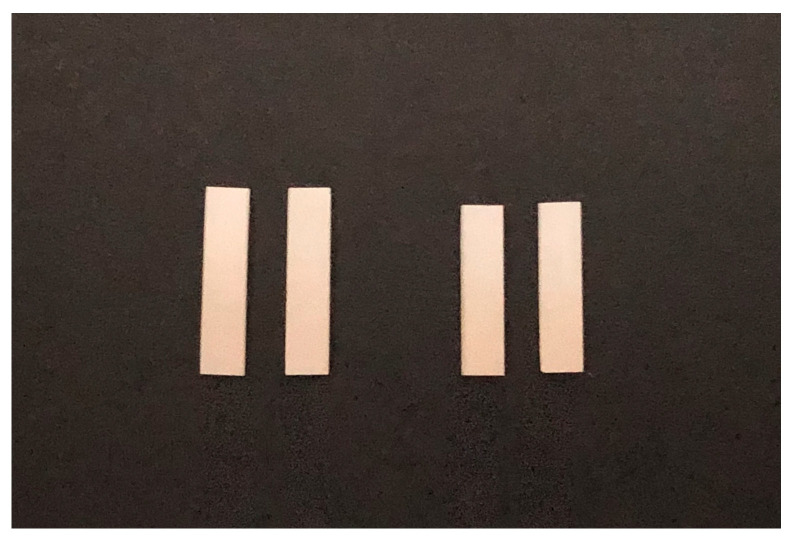
Shapes of sintered specimens: Katana STML (left)/Ceramill Zolid FX ML (right).

This study employed a 2 × 2 × 5 factorial design (two zirconia materials, two sintering speeds, five glazing conditions) with 20 treatment combinations (total sample size = 600, n = 30/group). Since 3-way ANOVA is a linear model, regression analysis can be used to determine if all effects explain a certain target proportion of the variance in the dependent variable [40]. Thus, the sample size was calculated using “Linear multiple regression: Fixed model, R^2^ deviation from zero” in software (G*Power Version 3.1, Heinrich-Heine-University, Düsseldorf, Germany) [40]. The estimated total sample size was 599. This sample size achieves 95% power to detect an effect size of 0.04 for group differences, with an α error of 0.05. Bar-shaped 5Y-zirconia specimens (Figure 1 and Figure 2) were fabricated from FX (Ceramill Zolid FX ML, Amann Girrbach AG, Koblach, Austria) and ST (Katana STML, Kuraray Noritake Dental, Tokyo, Japan) blocks for the 3-point flexural strength tests and microstructural and crystal structural observations.

The zirconia blocks were cut using a precision cutting machine (AMC-200, Fairworks, Seoul, Republic of Korea) equipped with a diamond wheel. The specimens were then ground and mirror-polished sequentially with 800-, 1200-, 2000-, and 3000-grit SiC abrasive papers, and their sizes were checked using micrometers (MDC-25PX, Mitutoyo, Kanagawa, Japan). All edges along the longitudinal axis were chamfered by approximately 0.1 mm (ISO 6872:2024).

Each specimen was sintered using either a speed sintering protocol or conventional sintering protocol in a sintering furnace (inLab Profire, Dentsply Sirona, Charlotte, NC, USA), according to the recommendations of the manufacturer (Table 1). FX takes 6.03 h for conventional sintering and 2.08 h for speed sintering. ST takes 6.97 h for conventional sintering and 0.89 h for speed sintering.

The specimens showed approximately 20% sintering shrinkage. The final dimensions (thickness × width × length) of the sintered specimens were 1.62 (±0.01) × 4.04 (±0.03) × 20 mm^3^ for FX and 1.63 (±0.02) × 4.06 (±0.03) × 22 mm^3^ for ST (ISO 6872:2024) [41]. The two zirconia products used have a 2 mm difference in length but were used as is to preserve the original multilayered structure; however, the final dimensions of both zirconia specimens met the ISO 6872:2024 standard (the ratio of thickness to length should be ≤0.1) [41].

The FX and ST specimens were divided into two main groups based on the sintering speed as follows: conventional sintering and speed sintering. After sintering, the specimens were further divided into five subgroups (n = 30/group) based on the glazing conditions as follows: nonglazed (NG; control group), glazed once (G1), glazed twice (G2), glazed once-simulated (GS1), and glazed twice-simulated (GS2). The zirconia glazing process involves applying glaze paste and firing (heat treatment). For the G1 group, this process was performed once, and twice for the G2 group. For the GS1 and GS2 groups, glazing was simulated by heat treating the samples once and twice, respectively, without applying glazing paste to exclude the effect of the increase in thickness owing to glazing layer. For the glazed specimens (G1, G2), a skilled technician carefully applied a thin and uniform layer of glazing paste (initial IQ LP NF, GC, Kasugai, Japan) to one side of each specimen using a ceramic brush. Following the instructions of the manufacturer (Table 2), glazing heat treatment was performed in a porcelain furnace (Multimat 2 Touch, Dentsply, Bensheim, Germany). The final thickness after glazing for the G1 and G2 specimens was 1.68 (±0.02) and 1.72 (±0.03) mm for FX, and 1.68 (±0.03) and 1.73 (±0.03) mm for ST, respectively. The increase in thickness after each cycle of glazing was almost identical for both FX and STML, averaging 0.05 (±0.007) mm.

### 2.2. Flexural Strength Tests and Weibull Analysis

**Figure 3:**
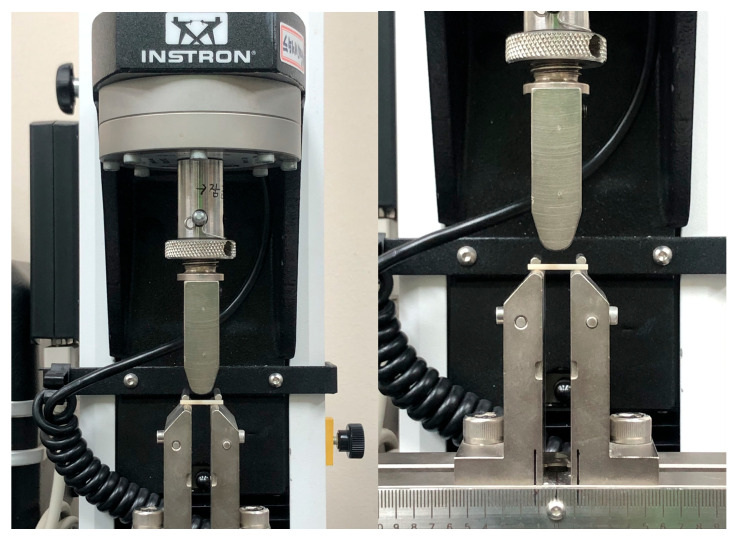
Images of specimen loading for flexural strength tests using the universal testing machine.

A universal testing machine (Instron 3345, Instron, Norwood, MA, USA) was used (Figure 3) for the 3-point flexural strength tests (n = 30/group), which were conducted according to ISO 6872:2024 [41]. The inter-support spacing was fixed at 12 mm, and a compressive force was applied vertically at a crosshead speed of 1 mm/min until fracture. The load value at fracture was measured with a software program (Bluehill2, Instron, Norwood, MA, USA). Glazing paste was applied to the compression side (top surface) to simulate the application of a glaze on the occlusal surface of a prosthesis. Flexural strength was calculated using the following equation [41]:

*σ* = 3*Nl*/2*bd*^2^

where *σ* refers to the flexural strength, *N* is the fracture load (N), *l* is the distance between supports (mm), *b* is the width, and *d* is the thickness of the specimen (mm).

Weibull analysis was performed on the flexural strength values. The Weibull modulus (*m*) and characteristic strength (*σ*_0_) were analyzed using the median ranking and maximum likelihood methods with a software program (Reliability and Maintenance Analyst Version 5.0.9, Engineered Software, Lacey, WA, USA). The Weibull distribution was characterized from the following equation [42]:

$$\mathrm{Pf}=1-\exp[-{\left(\frac{\sigma }{\sigma_{0}}\right)}^{m}]$$

where Pf is the fracture probability and equal to (rank − 0.3)/(N + 0.4), N is the number of samples, *σ* is the flexural strength, *σ*_0_ is the characteristic strength, and *m* is the Weibull modulus.

### 2.3. Field Emission Scanning Electron Microscopy (FE-SEM) Analysis

FE-SEM examination was performed to examine the microstructures (n = 2/group). As negligible compositional difference exists between each layer of zirconia used, FE-SEM examination was performed on the fractured specimens obtained after the flexural strength measurements without distinguishing between layers. A platinum coating was applied to the specimens for 60 s. Then, the specimens were observed under an FE-SEM instrument (JSM-7200F, Jeol, Akishima, Japan) at an accelerating voltage of 15 kV. The mean grain size was calculated from the FE-SEM images using the linear intercept method and a software program (ImageJ Version 1.53, National Institutes of Health, Bethesda, Rockville, MD, USA). Over 600 grains were used for each FX and ST specimen. The mean grain size (D) was determined using the following equation [11,43]:

D = 1.56*C*/*MN*

where *C* is the line length, *M* is the magnification of the micrograph, and *N* is the total count of intercepts. The applied correction factor was 1.56 [11,43,44].

### 2.4. X-ray Diffraction (XRD) Study

The crystal structure of the specimens was analyzed using high-resolution XRD (X’Pert^3^ Powder, PANalytical, Amsterdam, The Netherlands) with Ni-filtered CuKα radiation at 40 kV, 30 mA, and a step size of 0.013 degrees (n = 1/group). The central part of the long axis of the specimen was measured for XRD analysis. Rietveld refinement was performed on the obtained X-ray diffractograms by using a software program (Topas Academic Version 7.24, Bruker AXS, Karlsruhe, Germany). The fractions of tetragonal (*t*; space group, P4_2_/nmc), cubic (*c*; space group, Fm3m), and monoclinic (*m*; space group, P2_1_/c) phases were determined [45]. The standard Crystallographic Information Files (CIF) for the *t*-, *c*-, and *m*-phases were obtained from Lamas and Walsoe de Reca [45] and Howard et al. [46] in the Crystallography Open Database. Weighted Profile R-factor (Rwp), which is a crucial indicator of the goodness-of-fit in Rietveld refinement, was kept at below 10% to maintain the refinement quality. The yttria content (mol%) of obtained phase was determined from the analyzed lattice parameters (*a*, *c*) of the tetragonal phase using the following equations [47]:

$${\mathrm{YO}}_{1.5}\;(\mathrm{mol}\%)=\frac{1.0223-c/a\sqrt{2}}{0.001319}$$

$$Y_{2}O_{3}\;(\mathrm{mol}\%)=\frac{{YO}_{1.5}/100}{2-{YO}_{1.5}/100}\times 100$$

The total yttria content of the specimen was determined from the fraction of obtained phase (yttria-lean tetragonal (*t*) phase and yttria-rich tetragonal (*t*′) phase) and its yttria content using the following equation [48]:

$$\mathrm{Total}\;Y_{2}O_{3}\;(\mathrm{mol}\%)=\frac{t\;phase\left(wt\%\right)\times {Y_{2}O}_{3}\left(mol\%\right)\;in\;t\;phase}{100}+\frac{t^{\prime }phase\;\left(wt\%\right)\times {Y_{2}O}_{3}\left(mol\%\right)\;in\;t^{\prime }\;phase}{100}$$

### 2.5. Statistical Analysis

Statistical analysis (α = 0.05) was performed using a software program (IBM SPSS Statistics Version 25.0, IBM Corp, Armonk, NY, USA). The effects of sintering speed and glazing condition on the flexural strength of the zirconia specimens were analyzed using 3-way ANOVA and compared using the post hoc Scheffé test. The grain size was analyzed and compared using the Kruskal–Wallis H test followed by the Bonferroni–Dunn post hoc test.

## 3. Results

### 3.1. Flexural Strength and Weibull Modulus

The results of the three-way ANOVA showed that the flexural strength was significantly affected by the sintering speed, zirconia material, and glazing condition (*p* < 0.05, Table 3). No interaction was observed between these factors (*p* > 0.05).

As shown in Table 4, speed sintering had no significant effect on the flexural strength of the nonglazed (NG), glazed once and twice (G1, G2), and glazed once- and twice-simulated (GS1, GS2) groups of ST specimens compared with conventional sintering (*p* > 0.05). A similar trend was observed for the FX specimens (Table 4), except that the NG group showed a slight decrease in flexural strength following speed sintering (*p* < 0.05). Upon observing the effect of the zirconia material (Table 4), it was clear that FX generally exhibited higher flexural strength than ST, regardless of the sintering speed or glazing conditions. Glazing condition had a limited effect on flexural strength (Table 4); flexural strength differed significantly between the G2 and GS1 groups of conventionally sintered ST (*p* < 0.05). However, compared with the NG group, the G1, G2, GS1, and GS2 groups did not affect the flexural strength of both FX and ST significantly, regardless of the sintering speed (*p* > 0.05).

The Weibull analysis of the flexural strength of FX and ST (Table 5) showed similar Weibull modulus and characteristic strength across all groups, with overlapping ranges. The Weibull modulus values were in the range of 6.10 to 8.87 for FX and 6.51 to 8.14 for ST. The characteristic strength values were in the range of 626.02 to 704.78 MPa for FX and 539.66 to 618.76 MPa for ST.

### 3.2. Microstructure

**Figure 4:**
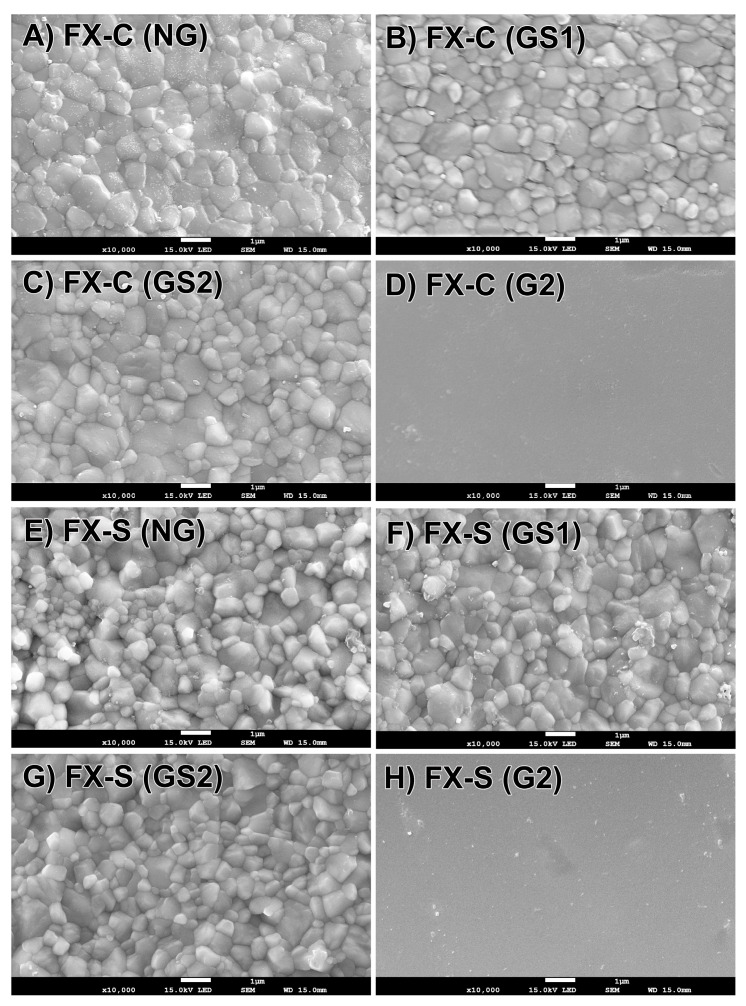
Microstructures of FX ML specimens (×10,000). NG, nonglazed; GS1, glazed once-simulated; GS2, glazed twice-simulated; G2, glazed twice.

**Figure 5:**
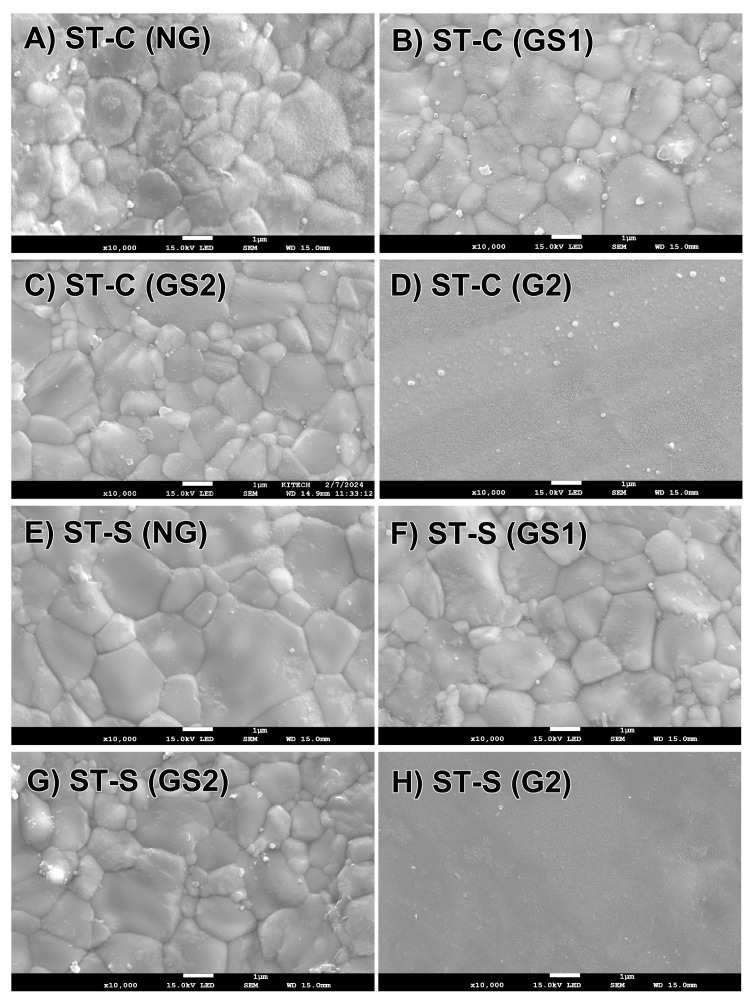
Microstructures of STML specimens (×10,000). NG, nonglazed; GS1, glazed once-simulated; GS2, glazed twice-simulated; G2, glazed twice.

As shown in Figure 4 for FX and Figure 5 for ST, the G2 groups (D,H) were covered with a glassy coating layer; thus, the microstructure and grain size following glazing were analyzed using the GS1 and GS2 groups.

For the FX specimens (Figure 4), the NG group (A,E) had similar equiaxed crystal structures at both sintering speeds; no change in these features was observed in the GS1 (B,F) and GS2 (C,G) groups. For the ST specimens (Figure 5), the NG group (A,E) showed small grains that were similar in size to the corresponding FX specimens and large grains at both sintering speeds; no change in these features was observed in the GS1 (B,F) and GS2 (C,G) groups.

Table 6 shows that speed sintering and glazing do not affect the grain size of FX or ST (*p* > 0.05). ST had a larger average grain size than FX in all groups (*p* < 0.05).

### 3.3. XRD Analysis

**Figure 6:**
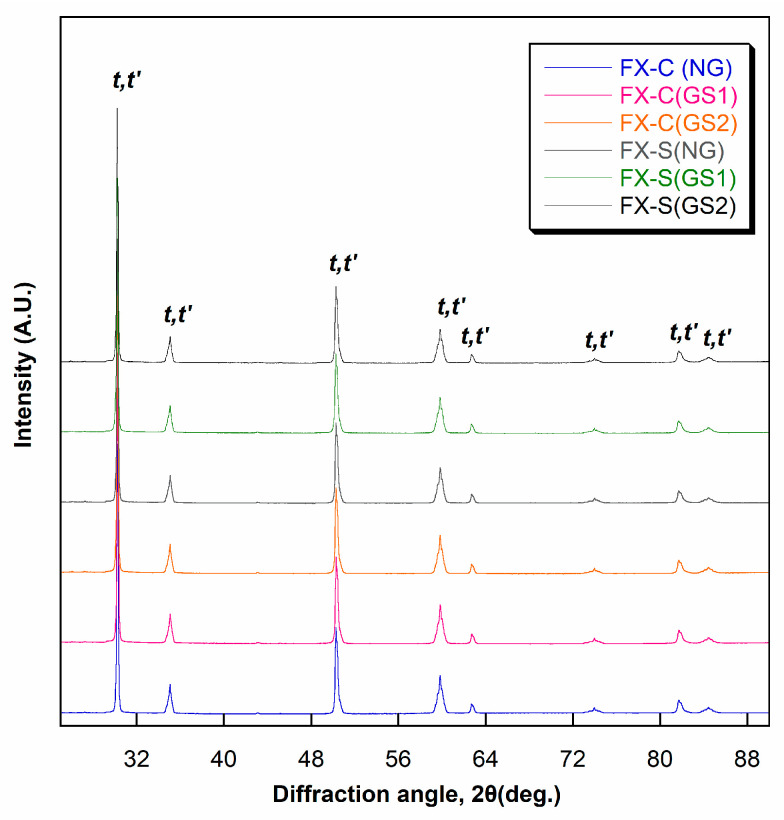
X-ray diffractograms for FX ML.

**Figure 7:**
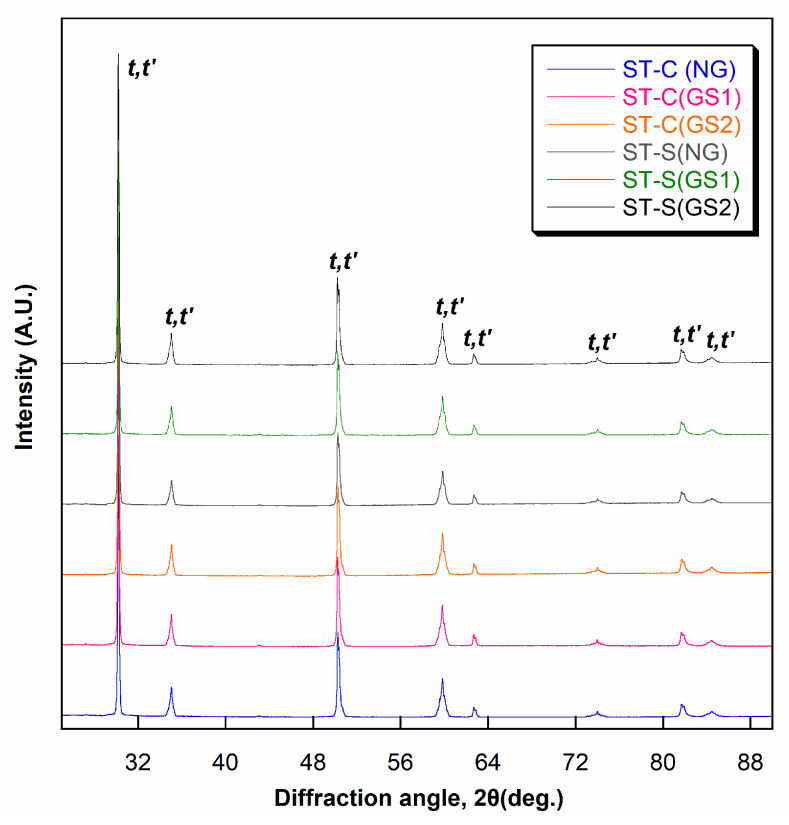
X-ray diffractograms for STML.

The X-ray diffractograms (Figure 6 and Figure 7) showed that the NG, GS1, and GS2 groups for FX and ST consisted of the yttria-lean tetragonal (*t*) phase and yttria-rich tetragonal (*t*′) phase after conventional and speed sintering. The results obtained from the Rietveld refinement (Table 7) showed that the phase fractions of *t*- and *t*′-phases remained almost unchanged even after two rounds of glazing after conventional and speed sintering. The phase fraction in FX was approximately 37% for the *t*-phase and 63% for the *t*′-phase. The phase fraction in ST was approximately 28% for the t-phase and 72% for the *t*′-phase. The tetragonality (axial ratio) and yttria content of the *t*- and *t*′-phases remained relatively constant across all experimental groups. The *t*′-phase consistently demonstrated a lower tetragonality and a higher yttria content compared with those of the *t*-phase across all experimental groups.

## 4. Discussion

The null hypothesis that speed sintering and glazing with and without glazing paste do not affect the flexural strength and microstructure of multilayered 5Y-zirconia was partially rejected. Sintering speed had a limited effect on flexural strength. Compared with the flexural strength observed after conventional sintering (NG; 657 ± 112 MPa), the flexural strength of FX decreased to 582 (±111) MPa after speed sintering (*p* < 0.05). While the manufacturer specified a flexural strength of 700 (±150) MPa for conventionally sintered FX, previous studies have reported lower values of 557 (±88) and 497 (±129) MPa [3,39]. Considering the large variation in these reported flexural strength values, the slight decrease in the flexural strength of FX owing to speed sintering could be considered clinically negligible. The ST specimens exhibited no difference in flexural strength after speed sintering. Similarly, the literature on speed-sintered ST reports no change in flexural strength after speed sintering at 1540 °C for 35 min or at 1560 °C for 30 min, even though the heating and cooling rates were lower than those in this study [9,25]. The flexural strength obtained in this study was within the range of flexural strength values reported for 5Y-zirconia (500 to 800 MPa) [3,22,25,27,39].

In a study on conventionally sintered 5Y-zirconia (preshade zirconia without multilayer), glazing did not affect flexural strength [39]. Similarly, in this study with multilayered 5Y-zirconia, glazing did not affect the flexural strength of both FX and ST, at both sintering speeds (*p* > 0.05). During flexural strength testing, failure typically occurs on the tensile side. However, in this study, glazing paste was applied to the compression side to simulate the application of a glaze on the occlusal surface of a prosthesis. Differences in the glazing application side (compression side, tensile side, and both sides) have been reported to have no effect on the flexural strength of zirconia [32]. A glazing layer thickness of 0.05 mm is known to be sufficient to maintain the durability of the glaze [35]. Because the increase in thickness by the glazing layer (approximately 0.05 mm per glazing in this study) was included in the specimen thickness for the flexural strength test, a glazing simulation without glazing paste was performed to exclude the effect of the increase in thickness owing to glazing layer. The results for both FX and ST showed no statistically significant difference in flexural strength between the G1, G2, GS1, and GS2 groups at both sintering speeds (except for the G2 and GS1 groups of conventionally sintered ST (*p* < 0.05)); thus, the increase in specimen thickness owing to the formation of a glazing layer up to approximately 0.1 mm had no or minimal effect on the flexural strength. In the case of 3Y-TZP, glazing without a glazing material did not affect the flexural strength of the zirconia, as observed in this study; however, applying the glazing material to the zirconia specimens (resulting in a 0.1 mm increase in thickness) led to a significant decrease in flexural strength [36]. These discrepancies could be attributed to the use of different zirconia and glazing materials or variations in the glazing heat treatment process.

The Weibull characteristic strength represents the strength at which 63.2% of the specimens are expected to fail [42]. Regardless of the sintering speed, the characteristic strength of both FX and ST was slightly lower in the G2 group than in the G1 group. However, the values were comparable, with overlapping ranges. A similar trend was observed for the GS1 and GS2 groups. The Weibull modulus indicates the reliability of flexural strength, with higher values reflecting a higher reliability of the flexural strength of a material [42]. The Weibull modulus values for FX and ST remained relatively unchanged after speed sintering and glazing, and ranged from 6.10 to 8.87 for FX and 6.51 to 8.14 for ST. These values correspond to those reported for conventionally sintered FX and ST [3,17].

The yttria-gradient 5Y/4Y-zirconia (e.max MT Multi) with 5Y-zirconia in the enamel layer and 4Y-zirconia in the dentin layer exhibited a microstructure gradient with decreasing grain size from the enamel layer to the dentin layer in the conventionally sintered state [49]. By contrast, the multilayer zirconia Katana STML (ST), which has a uniform yttria content across all layers [11], exhibited no differences in microstructure and grain size between layers in the conventionally sintered state [49]. In this study, the microstructure of ST was analyzed randomly without layer distinction, but the resulting grain size was very similar to that analyzed layer-by-layer [49]. Analysis of grain size according to sintering speed showed that for FX and ST, there was no significant difference in grain size between conventional and speed-sintered specimens. Furthermore, even after two rounds of glazing (simulation) following speed sintering, there was no significant difference in grain size compared to conventionally sintered and then glazed specimens. Similarly in the literature, multilayered 5Y-zirconia (Cercon xt ML, Lava Esthetic) showed a minor decrease in grain size after speed sintering compared to conventional sintering, where the grain size was not different across all layers [43]. The absence of a significant difference in grain size between conventional and speed-sintered specimens was also reported for 4Y-zirconia (Zolid Gen-X) and 6Y-zirconia (Katana UTML) [24]. In this study, ST had a larger average grain size than FX in most groups (*p* < 0.05). Grain size in dental zirconia is known to affect flexural strength [20,21]. In previous studies on 3Y-TZP (Ceramill ZI, Zpex), an increase in grain size by sintering at a higher temperature or for longer time caused a decrease in flexural strength [20,21]. Similarly, ST, which had a larger average grain size than FX had a lower flexural strength than FX in most groups (*p* < 0.05).

In this study on two 5Y-zirconia materials, a minimal change in the phase fractions was observed even after two rounds of glazing (simulation) after conventional and speed sintering. In the literature, speed-sintered ST without glazing showed comparable results; in a study in which ST was speed sintered with slightly different protocols, no change in phase fractions and flexural strength was observed [17]. In another study, speed-sintered ST exhibited negligible differences in phase fraction compared to conventionally sintered specimens, and no change in Weibull characteristic strength was observed [9].

Dental zirconia products containing 4 to 6 mol% yttria are widely known to be composed of the cubic (*c*) and tetragonal (*t*) phases [9,10,11,17]. However, recent research has revealed that 4Y- to 6Y-zirconia has an yttria-rich tetragonal (*t*′) phase instead of the *c*-phase [47,48]. These studies either reported a mismatch in the total yttria content, attributed to the presence of the *c*-phase, as determined by Rietveld analysis of X-ray diffractograms in conjunction with the phase equilibrium diagram [47], or reported the absence of *c*-phase peaks in high-resolution X-ray diffractograms obtained by fine scanning within the 2θ range of 72–76 degrees, following the elimination of Kα_2_ radiation using a monochromator [48]. The yttria-rich *t*′-phase has an axial ratio very similar to that of the *c*-phase, thus it has less light birefringence than the general *t*-phase, contributing to the improvement of translucency [50]. In this study, the obtained phase fraction in all ST groups was approximately 28% for the *t*-phase and 72% for the *t*′-phase. These values agreed well with the values for the conventionally sintered ST in the latest literature [48], where the total yttria value was revealed to be 5.83 (mol%) by using X-ray fluorescence spectroscopy [48]. This finding is consistent with our results for the total yttria value of ST. Unlike the *t*-phase, the *t*′-phase does not undergo a stress-induced martensitic transformation, that is, transformation toughening [50]. In this study, ST had a lower flexural strength than FX in most groups (*p* < 0.05), which was attributed to the lower *t*-phase content as well as greater grain size than FX [21,50].

This study is limited by the use of only two zirconia materials and two sintering speeds. The conclusions drawn might not be applicable to other types of zirconia materials or sintering protocols. Further studies with broader material sets and sintering parameters are needed. This study lacks data on the long-term performance, aging, and durability under cyclic loading of zirconia, which are crucial for dental materials. Additionally, this study did not perform a fracture analysis of zirconia. However, to understand and improve the properties of zirconia, aging, durability under cyclic loading, fracture surface morphology, and crack pattern need to be further investigated.

## 5. Conclusions

Highly translucent multilayered 5Y-zirconia is considered suitable for dental applications using speed sintering and subsequent glazing, demonstrating minimal or no change in flexural strength and microstructure in the as-sintered and glazed states.

## Figures and Tables

**Table 1:** Zirconia sintering conditions.

Zirconia Material	Sintering Rate	Code	Stage	Heating and Cooling Rate (°C/min)	Temp. (°C)	Holding Time (min)
Ceramill Zolid FX ML	Conventional	FX-C	1	8	1450	120
			2	−20	200	0
	Speed	FX-S	1	60	980	0
			2	12	1350	0
			3	15	1450	65
			4	−120 *	700	0
Katana STML	Conventional	ST-C	1	10	1550	120
			2	−10	100	0
	Speed	ST-S	1	120	1450	0
			2	10	1600	20
			3	−120	800	0

* The manufacturer-provided cooling condition is 70% door opening; this condition was modified to unify the sintering furnace in this study.

**Table 2:** Glazing heat treatment program.

Start Temp. (°C)	Dry (min)	Heating Rate (°C/min)	Final Temp. (°C)	Holding Time (min)	Vacuum
480	2	45	820	1	-

**Table 3:** Outcomes of three-way ANOVA on flexural strength.

Variation Source	Type III Sum of Squares	Degree of Freedom	Mean Square	F-Statistic	*p*-Value
Corrected model	1,229,372.482 ^a^	19	64,703.815	7.138	<0.001
Intercept	198,477,217.375	1	198,477,217.375	21,896.561	<0.001
S	222,562.786	1	222,562.786	24.554	<0.001
Z	772,500.827	1	772,500.827	85.224	<0.001
G	138,196.874	4	34,549.218	3.812	0.005
S × Z	19,227.619	1	19,227.619	2.121	0.146
S × G	49,086.356	4	12,271.589	1.354	0.249
Z × G	19,155.271	4	4788.818	0.528	0.715
S × Z × G	20,474.150	4	5118.537	0.565	0.688
Error	5,329,814.366	588	9064.310		
Total	205,665,734.551	608			
Corrected total	6,559,186.848	607			

^a^ R squared = 0.189 (adjusted R squared = 0.162); S, sintering speed; G, glazing conditions; Z, used zirconia material.

**Table 4:** Mean flexural strength (MPa) ± standard deviation for different zirconia groups.

Code	NG	G1	G2	GS1	GS2
**FX-C**	657.35 ^Ac^	647.77 ^Ab^	593.30 ^Ab^	642.44 ^Ab^	622.11 ^Ab^
	(112.02)	(94.03)	(106.61)	(112.88)	(109.59)
**FX-S**	581.59 ^Ab^	588.01 ^Aab^	558.52 ^Aab^	583.04 ^Aab^	603.93 ^Ab^
	(111.02)	(99.37)	(105.21)	(105.81)	(94.69)
**ST-C**	564.12 ^ABab^	542.25 ^ABa^	505.70 ^Aa^	579.41 ^Bab^	558.26 ^ABab^
	(82.87)	(79.94)	(88.27)	(94.49)	(93.16)
**ST-S**	507.93 ^Aa^	530.66 ^Aa^	513.54 ^Aa^	540.43 ^Aa^	521.92 ^Aa^
	(75.30)	(91.97)	(77.46)	(74.56)	(73.61)

NG, nonglazed; G1, glazed once; G2, glazed twice; GS1, glazed once-simulated; GS2, glazed twice-simulated; FX-C, conventionally sintered FX; FX-S, speed-sintered FX; ST-C, conventionally sintered ST; ST-S, speed-sintered ST. No statistically significant differences exist among groups sharing the same uppercase letter in a row, and no statistically significant differences exist among groups sharing the same lowercase letter in a column.

**Table 5:** Weibull modulus (*m*) and characteristic strength (*σ*_0_) for different zirconia groups.

Code	*m*	NG	G1	G2	GS1	GS2
	*σ*_0_ (MPa)					
**FX-C**	***m***	6.42 (4.89–8.42)	8.87 (6.61–11.89)	6.78 (5.11–9.01)	6.63 (4.99–8.81)	6.63 (5.02–8.75)
	***σ*_0_**	704.78 (664.33–747.70)	686.36 (657.88–716.08)	635.92 (601.58–672.22)	689.49 (651.25–729.98)	666.87 (629.92–705.98)
**FX-S**	***m***	6.10 (4.61–8.07)	7.66 (5.87–9.98)	6.26 (4.84–8.10)	6.65 (5.15–8.58)	8.32 (6.39–10.83)
	***σ*_0_**	626.43 (588.84–666.43)	656.94 (628.92–686.21)	638.02 (604.78–673.09)	626.02 (595.25–658.38)	667.46 (641.19–694.80)
**ST-C**	***m***	7.99 (6.03–10.56)	7.41 (5.67–9.68)	6.51 (4.94–8.57)	7.04 (5.35–9.27)	6.91 (5.26–9.08)
	***σ*_0_**	599.09 (571.40–628.11)	576.50 (547.75–606.76)	542.34 (511.72–574.79)	618.76 (586.41–652.89)	596.61 (564.89–630.11)
**ST-S**	***m***	8.11 (6.09–10.81)	7.49 (5.56–10.08)	7.99 (6.03–10.61)	8.14 (6.22–10.64)	7.82 (5.97–10.26)
	***σ*_0_**	539.66 (515.12–565.36)	567.22 (539.50–596.35)	545.73 (520.60–572.07)	572.12 (546.13–599.35)	553.81 (527.62–581.30)

NG, nonglazed; G1, glazed once; G2, glazed twice; GS1, glazed once-simulated; GS2, glazed twice-simulated; FX-C, conventionally sintered FX; FX-S, speed-sintered FX; ST-C, conventionally sintered ST; ST-S, speed-sintered ST.

**Table 6:** Average grain size for different zirconia groups.

Code	Grain Size (μm)	NG	GS1	GS2
**FX-C**	M	0.871 ^Aa^	0.818 ^Aa^	0.869 ^Aa^
	±SD	(0.230)	(0.197)	(0.199)
**FX-S**	M	0.850 ^Aa^	0.831 ^Aa^	0.810 ^Aa^
	±SD	(0.112)	(0.174)	(0.142)
**ST-C**	M	1.660 ^Ab^	1.786 ^Ab^	1.705 ^Ab^
	±SD	(0.109)	(0.128)	(0.199)
**ST-S**	M	1.679 ^Ab^	1.778 ^Ab^	1.705 ^Ab^
	±SD	(0.123)	(0.186)	(0.139)

NG, nonglazed; GS1, glazed once-simulated; GS2, glazed twice-simulated; FX-C, conventionally sintered FX; FX-S, speed-sintered FX; ST-C, conventionally sintered ST; ST-S, speed-sintered ST. No statistically significant differences exist among groups sharing the same uppercase letter in a row, and no statistically significant differences exist among groups sharing the same lowercase letter in a column.

**Table 7:** Zirconia phase fractions for various zirconia groups.

		Phase Fraction (wt%)		Tetragonality (Axial Ratio)		Y_2_O_3_ Content (mol%)		
		*t*	*t*′	*t*	*t*′	*t*	*t*′	Total
**FX-C**	NG	37.6	62.4	1.0147	1.0043	2.96	7.33	5.68
	GS1	37.8	62.2	1.0147	1.0042	2.97	7.36	5.70
	GS2	37.1	62.9	1.0149	1.0046	2.90	7.25	5.64
**FX-S**	NG	37.2	62.8	1.0143	1.0046	3.13	7.19	5.68
	GS1	36.9	63.1	1.0141	1.0043	3.19	7.30	5.78
	GS2	37.5	62.5	1.0142	1.0046	3.15	7.21	5.69
**ST-C**	NG	28.2	71.8	1.0156	1.0045	2.61	7.23	5.92
	GS1	27.9	72.1	1.0158	1.0047	2.53	7.16	5.86
	GS2	28.7	71.3	1.0156	1.0046	2.62	7.19	5.87
**ST-S**	NG	27.2	72.8	1.0149	1.0049	2.90	7.04	5.91
	GS1	28.5	71.5	1.0147	1.0046	2.96	7.20	5.99
	GS2	27.7	72.3	1.0145	1.0049	3.03	7.06	5.94

NG, nonglazed; GS1, glazed once-simulated; GS2, glazed twice-simulated; FX-C, conventionally sintered FX; FX-S, speed-sintered FX; ST-C, conventionally sintered ST; ST-S, speed-sintered ST.

## Data Availability

The datasets generated and analyzed in this study are available in the article. Further inquiries can be directed to the corresponding author.
